# The fiscal cost of weak governance: Evidence from teacher absence in India

**DOI:** 10.1016/j.jpubeco.2016.11.005

**Published:** 2017-01

**Authors:** Karthik Muralidharan, Jishnu Das, Alaka Holla, Aakash Mohpal

**Affiliations:** a University of California, San Diego, CA, United States; b The World Bank Group, Washington, DC, United States; c University of Michigan, Ann Arbor, MI, United States

**Keywords:** Education, Teacher absence, Teacher absenteeism, India, Governance, State capacity, Monitoring

## Abstract

The relative return to strategies that augment inputs versus those that reduce inefficiencies remains a key open question for education policy in low-income countries. Using a new nationally-representative panel dataset of schools across 1297 villages in India, we show that the large public investments in education over the past decade have led to substantial improvements in input-based measures of school quality, but only a modest reduction in inefficiency as measured by teacher absence. In our data, 23.6% of teachers were absent during unannounced school visits, and we estimate that the salary cost of unauthorized teacher absence is $1.5 billion/year. We find two robust correlations in the nationally-representative panel data that corroborate findings from smaller-scale experiments. First, reductions in student-teacher ratios are correlated with increased teacher absence. Second, increases in the frequency of school monitoring are strongly correlated with lower teacher absence. Using these results, we show that reducing inefficiencies by increasing the frequency of monitoring could be over ten times more cost effective at increasing the *effective* student-teacher ratio than hiring more teachers. Thus, policies that decrease the inefficiency of public education spending are likely to yield substantially higher marginal returns than those that augment inputs.

## Highlights

• A nationwide representative sample of public schools in rural India found teacher absence rate of 23.6%.
• Increases in school monitoring were strongly correlated with reductions in teacher absence rates.
• Investing in reducing teacher absence through better monitoring could be over ten times more cost effective at reducing the effective student teacher ratio (net of teacher absence) than investing in hiring more teachers.
• The marginal returns to inefficiency reduction are likely to be considerably higher than the returns to augmenting school inputs in a “business as usual” manner.

## 1 Introduction

Determining the optimal level and composition of public education spending is a key policy question in most low-income countries. Many education advocates believe that low-income countries need substantial increases in public education spending to meet enrollment and learning goals (UNESCO, 2014); others argue that public sector inefficiencies leave considerable room for improvement within existing education budgets, and that fiscal constraints make it imperative to improve the efficiency of public expenditure (World Bank, 2010). However, the data to assess the relative importance of these contentions remains sparse, in part, due to the difficulty in detecting and measuring inefficiencies in public spending.

In this paper, we study one striking measure of public sector inefficiency - teacher absences - with panel data collected 7  years apart in India at a time of sharp increases in education spending. A large portion of this increase was accounted for by the salary cost of hiring teachers to reduce the student-teacher ratio in public schools. As a policy alternative to hiring more teachers, we show that reducing teacher absences by increasing school monitoring could be over ten times more cost effective at reducing the *effective* student-teacher ratio (net of teacher absence). Thus, while the default approach to improving education in low-income countries is input-augmentation, our results suggest that investing in reducing inefficiencies may yield much greater returns.

India presents a particularly salient setting for our analysis. It has the largest primary education system in the world, catering to over 200 million children. Further, over the past decade, the Government of India has invested heavily in primary education under the Sarva Shiksha Abhiyan (SSA) or “Education for All Campaign.” Partly financed by a special education tax, this national program sought to correct historical inattention to primary education and led to a substantial increase in annual spending on primary education across several major categories of inputs including school infrastructure, teacher quality, student-teacher ratios, and school feeding programs.^1^

However, the public education system in India also faces substantial governance challenges that may limit the extent to which this additional spending translates into improved education outcomes. Our indicator of systemic inefficiency - teacher absence - presents a particularly striking indicator of weak governance. A nationally-representative study of over 3000 public primary schools across 19 major Indian states found that over 25% of teachers were absent from work on a typical working day in 2003 (Kremer et al., 2005). Although administrative data from the government's official records suggest that SSA has led to an improvement in various input-based measures of school quality, there is little evidence on whether these investments have translated into improvements in education system performance, both with respect to intermediate metrics such as teacher absence and final outcomes such as test scores.^2^

Our study of this nationwide campaign to improve school quality in India uses a new nationally-representative panel dataset of education inputs and outcomes that we collected in 2010. We constructed this dataset by revisiting a randomly-sampled subset of the villages originally surveyed in 2003 (see Kremer et al. (2005)) and collecting detailed data on school facilities, teachers, community participation, monitoring visits by officials, and teacher absence rates. Thus, in addition to reporting updated estimates of teacher absence, and independently-measured summary statistics on input-based measures of school quality, we are able to correlate changes in input-based measures of school quality with changes in teacher absence. The panel data help mitigate concerns arising from fixed unobserved heterogeneity at the village-level, and let us study how the sharp increases in public education spending over the last decade have affected school quality.

We find significant improvements in almost all input-based measures of school quality between 2003 and 2010. The fraction of schools with toilets and electricity more than doubled, and the fraction serving mid-day meals nearly quadrupled. There were significant increases in the fraction of schools with drinking water, libraries, and a paved road nearby. The fraction of teachers with college degrees increased by 41%, and student-teacher ratios (STR) fell by 16%. The fraction of teachers not paid on time fell from 51 to 22%, and the fraction of teachers reporting the existence of teacher recognition programs increased from 50 to 81%. Finally, the frequency of school inspections and parent-teacher association (PTA) meetings increased significantly.

However, reductions in teacher absence rates were more modest. The all-India weighted average teacher absence in rural areas fell from 26.3 to 23.6%. 3 While increased teacher hiring brought the STR down from 47 to below 40, the *effective* STR (ESTR), after accounting for teacher absences was still over 50 (having reduced from 64 in 2003 to 52 in 2010). The variation in teacher absence across states remains high. At one end, several top performing states have teacher absence rates below 15%, while at the other end, the poorest performing state, Jharkhand, has a teacher absence rate of 46%.

Our panel-data analysis, where we correlate changes in village-level teacher absence with changes in teacher and school characteristics, and administrative and community-level monitoring, yields two robust correlations. First, reductions in the school-level student-teacher ratio (STR) are correlated with an *increase* in teacher absence, suggesting that the potential benefits from investing in more teachers and lower STR may be partly offset by an increase in teacher absence. Second, better top-down administrative monitoring is strongly correlated with lower teacher absence. Absence rates were 6.5 percentage points lower in villages with regular public school inspections relative to those without, which is a 25% reduction in overall absence and a 40% decline in unauthorized absence.^4^

One way to estimate the cost of teacher absence is to calculate the salary cost paid by the government to teachers for days of work that they did not attend. We estimate this fiscal cost to be over $1.5 billion per year, which is around 60% of the entire revenue collected from the special education tax used to fund SSA in 2010.^5^ Teacher salaries typically account for over 80% of non-capital education spending (Dongre et al., 2014), and the most expensive component of the recently passed Right to Education (RtE) Act in India is a commitment to reduce STR from 40:1 to 30:1, by hiring more teachers at an additional cost of $5  billion/year. Using the most conservative panel-data estimates of the correlations between increased monitoring and reduced teacher absence, we estimate that improving school governance (by hiring more supervisory staff) could be over *ten times* more cost effective at increasing *effective* student-teacher ratio (net of teacher absence) than hiring more teachers. These calculations suggest that the marginal returns to investing in an inefficiency-reduction strategy (through better monitoring and governance of the education system) are likely to be much higher than a typical input-augmentation strategy.

This paper makes several contributions to the literature on public economics in low-income countries. First, teacher absence is now widely used as a governance indicator in education in low- and middle-income countries.^6^ We update estimates of teacher absence in rural India from 2003 and show that despite substantial increases in education spending over the last decade, improvements on this key measure of governance have been more modest. While corruption in education spending has been shown to hurt learning outcomes (Ferraz et al., 2012), our results highlight the importance of also focusing on governance issues that lead to significant amounts of ‘passive’ waste and inefficiency on an ongoing annual basis, but may not obtain as much media attention as one-off corruption scandals (Bandiera et al., 2009, World Bank, 2010).

Second, the fact that decreases in STRs are correlated with *increased* teacher absence underscores the importance of distinguishing between average and marginal rates of corruption and waste in public spending. Niehaus and Sukhtankar (2013) propose this terminology in the context of wages paid in a public-works program in India and find that marginal rates of leakage are much higher than average rates. We find the same result in the context of teachers and show that the effective absence rate of the marginal teacher hired is considerably higher than the average absence (because of the increased absence among existing teachers). This result, from a large all-India sample, mirrors smaller-sample experimental findings in multiple settings. Duflo et al. (2015), and Muralidharan and Sundararaman (2013) present experimental evidence (from Kenya and India) showing that provision of an extra teacher to schools led to an increase in the absence rate of existing teachers in *both* settings. In other words, additional spending on school inputs (of which teacher salaries are the largest component) was correlated with increased inefficiency of spending.

Third, improvements in top-down administrative monitoring (inspections) are more strongly correlated with reduced teacher absence than improvements in bottom-up community monitoring (PTA meetings), consistent with experimental evidence on the relative effectiveness of administrative and community audits on reducing corruption in road construction in Indonesia (Olken, 2007). More broadly, a growing body of experimental evidence points to the effectiveness of audits and monitoring (accompanied by rewards or sanctions) in improving the performance of public-sector workers and service providers (including Olken (2007) in Indonesia; Duflo et al. (2012) in India; and Zamboni and Litschig (2016) in Brazil). Our panel-data estimates using data from an “as is” nationwide increase in monitoring of schools provide complementary evidence to smaller-scale experiments and suggest that investing in better governance and monitoring of service providers may be an important component of improving state capacity for service delivery in low-income countries (Besley and Persson, 2009, Muralidharan et al., 2016).

Finally, recent research has pointed to ‘misallocation’ of capital and labor in low-income countries as an important contributor to lower total factor productivity (TFP) in these settings (Hsieh and Klenow, 2009), and has also documented that a plausible reason for this misallocation is that ‘management quality’ is poorer in low-income countries, and that public-sector firms are managed especially poorly (Bloom and Van Reenen, 2010). Our results provide a striking example of weak management and misallocation in publicly-produced primary education in India (a sector that accounts for over 3% of GDP in spending). In particular, our estimates suggest that reallocating a portion of the $5 billion/year increase in education spending budgeted for hiring more teachers towards measures focused on reducing teacher absence (for instance, by hiring more supervisory staff) may be a much more cost effective way of increasing effective teacher-student contact time. Thus, misallocation is likely to be a first-order issue in this setting, and reallocating education spending towards better governance may substantially increase TFP in publicly-produced education.^7^

The rest of this paper is organized as follows: Section 2 discusses our empirical methods and analytical framework. Section 3 reports summary statistics on school inputs and teacher absence. Section 4 presents the cross-sectional and panel regression results. Section 5 discusses the fiscal costs of weak governance and compares the returns to investing in better monitoring with that from hiring more teachers. Section 6 discusses policy implications, and Section 7 concludes.

## 2 Data and analytic framework

The nationally-representative sample used for the 2003 surveys, which our current study uses as a base, covered both urban and rural areas across the 19 most populous states of India, except Delhi. This represented over 95% of the country's population. The 2010 sample covered only *rural* India. The sampling strategy in 2010 aimed to maintain representativeness of the current landscape of schools in rural India, and to maximize the size of the panel. We met these twin objectives by retaining the villages in the original sample to the extent possible, while re-sampling schools from the full universe of schools in these villages in 2010, and conducting the panel analysis at the village level.^8^

Enumerators first conducted school censuses in each village, from which we sampled up to three schools per village for the absence surveys. During fieldwork, enumerators made three separate visits to each sampled school over a period of 10 months from January–October 2010.^9^ Data on school infrastructure and accessibility, finances, and teacher demographics were collected once for each school (typically during the first visit, but completed in later visits if necessary), while data on time-varying metrics such as teacher and student attendance and dates of the most recent inspections and PTA meetings were collected in each of the three visits. We also assessed student learning with a test administered to a representative sample of fourth grade students in sampled schools. See Appendix A, Table A1 – A3 for further details on sampling and construction of the village-level panel data set.

Teacher absence was measured by direct physical verification of teacher presence within the first fifteen minutes of a survey visit. Data collected during the school census were used to pre-populate teacher rosters for the sampled schools, so that enumerators could look for teachers and record their attendance and activity immediately after their arrival at the school.^10^ Once teacher attendance was recorded, all other data were collected using interviews of head teachers and individual teachers.^11^

We record teachers as absent on a given visit if they were not found anywhere in the school in the first fifteen minutes after enumerators reached a school. We consider all the teachers in the school to be absent if the school was closed during regular working hours on a school day, and respondents near the school did not know why the school was closed or mentioned that the school was closed because no teacher had arrived or they had all left early.^12^ To be conservative in our measure of absence, we exclude all school closures due to bad weather, school construction/repairs, school functions and alternative uses of school premises (for instance, elections). We also exclude all part-time teachers, teachers who were transferred or reassigned elsewhere, or teachers reportedly on a different shift.

We construct a school infrastructure index by adding binary indicators for the presence of drinking water, toilets, electricity, and a library. We construct a remoteness index by taking the average of nine normalized indicators of distance to various amenities including a paved road, bus station, train station, public health facility, private health clinic, university, bank, post-office and Ministry of Education office. A lower score on the remoteness index represents a better connected school. During each survey visit, enumerators referred to written school records to note the date of the most recent school inspection, and the date of the most recent parent-teacher association (PTA) meeting. Average parental education of children in a school is computed from the basic demographic data collected for the sample of fourth-grade students chosen for assessments of learning outcomes.

For most of the analysis in this paper, we use the village as our unit of analysis and examine mean village-level indicators of both inputs and outcomes because a large number of new schools had been constructed between 2003 and 2010, including in villages that already had schools. This school construction resulted from a policy designed to improve school access by ensuring that every habitation with over 30 school-age children had a school within a distance of one kilometer. Thus, to ensure that our sample was representative in 2010, and at the same time amenable to panel data analysis relative to 2003, we constructed the panel at the village level, with a new representative sample of schools drawn in the sampled villages.^13^ All the results reported in this paper are population weighted and are thus representative of the relevant geographic unit (i.e., state or all-India).

## 3 Summary statistics

### 3.1 Changes in inputs

The data show considerable improvements in school inputs between 2003 and 2010 along three broad categories - teacher qualifications and working conditions, school facilities, and monitoring (Table 1 - Panels A–C). The fraction of teachers with a college degree increased from 41 to 58%, the fraction reporting that they were getting paid regularly rose from 49 to 78%, and the fraction reporting the existence of teacher recognition schemes rose from 50 to 81%. The fraction of teachers who report a formal teaching credential fell from 77 to 68%, largely due to a significant increase in the hiring of contract teachers (who are not required to have teaching credentials) in several large states. In our data, the fraction of teachers on a temporary contract or ‘contract teachers' increased from 6 to 30%.

School facilities and infrastructure improved on almost every measure. The fraction of schools with toilets and electricity more than doubled (from 40% to 84% for toilets and 22% to 45% for electricity); the fraction of schools with functioning mid-day meal programs nearly quadrupled (from 22% to 79%); the fraction of schools with a library increased by over 35 %(from 51% to 69%), and almost all schools now have access to drinking water (96%). Initiatives outside the education ministry to increase road construction have also led to increased proximity of schools to paved roads increasing the accessibility of schools for teachers who choose to live farther away. Relative to the distribution observed in 2003, a summary index of school infrastructure improved by 0.9 standard deviations.^14^

We also find improvements in both ‘top-down’ administrative and ‘bottom-up’ community monitoring of schools over this period. The fraction of schools inspected in the three months prior to a survey visit increased from 38% to 56%. The extent of community oversight of schools, measured by the frequency of PTA meetings also increased: The probability that a PTA meeting took place during the three months prior to a survey visit increased from 30% to 45%. Overall, Table 1 (Panel A–C) confirms that the Government of India's increased focus on primary education in the past decade did lead to significant improvements in input-based measures of school quality, as well as administrative and community monitoring.

### 3.2 Changes in teacher absence

We now turn to changes in teacher absence. Table 1 (Panel D) shows that the population-weighted national average teacher absence rate for rural India fell from 26.3 percent% to 23.6%, a reduction of 10%. Since students receive reduced teacher attention when teachers are absent, we divide the STR by “1 - teacher absence rate” to obtain the *effective* student teacher ratio (ESTR). Although the all-India STR had been reduced to below 40 in this period, the effective STR after accounting for teacher absence was still over 52. We present state-level data on teacher absence rates and ESTR for 2003 and 2010 in Table A4.^15^

Chaudhury et al. (2006) find a strong negative correlation between GDP/capita and teacher absence rates (both across countries and within Indian states). Hence, one way to interpret the magnitude of these changes is to compare them with the expected reduction in teacher absence that may be attributed simply to the economic growth that has taken place in this period. Using a growth accounting (as opposed to causal) framework, we can decompose the change in teacher absence into a component explained by changes in GDP/capita (as a proxy for ‘inputs') and one explained by a change in governance (a proxy for TFP). Cross-sectional estimates from the 2003 data suggest that a 10 percent increase in GDP/capita is associated with a 0.6 percentage point reduction in teacher absence.^16^ In the period between 2002 and 2010, real GDP/capita in India had grown by 38%. Thus, growth in GDP/capita over this period should have by itself contributed to a reduction in teacher absence of 2.4%. Our estimate of the change in teacher absence rate is exactly in this range, and suggests that the reduction of teacher absence we document is consistent with a proportional increase in ‘inputs' into education, but a limited improvement in TFP in this period. We discuss the policy implications of this result in the conclusion.

### 3.3 Stated reasons for absence, teaching activity, and official records

In cases where a teacher was not found in the school, enumerators asked the head teacher (or senior-most teacher present) for the reason for absence. These stated reasons are summarized in Table 2 (Panel A). Two categories of clearly unauthorized absence (school closure during working hours and no valid reason for absence) account for just under half the cases of teacher absence (48%), which provides a lower bound on the extent of unauthorized absences of 11.3 percentage points. The two other categories of stated absence (authorized leave and official duties) that account for 52% of the observed absence are potentially legitimate but cannot be verified.

While head teachers may overstate the extent of official duties to shield absent colleagues, they should have no reason to understate it. We can, therefore, reasonably treat the stated reasons for absence as an upper bound for duty-induced absence. This yields the important finding that one commonly cited reason for teacher absence - namely, that teachers are often asked to perform non-teaching duties such as conducting censuses and monitoring elections - is a very small contributor to the high rate of observed teacher absence. Table 2 - Panel A shows that official non-teaching duties account for less than 1% of observations and under 4% of the cases of teacher absence (these results are unchanged from 2003).

In cases where the teacher was present, enumerators recorded the activity that the teacher was engaged in at the point of observation: 53% of teachers on the payroll were found to be actively teaching, and another 4% were coded as passively teaching (defined as minding the class while students do their own work). Just over 19% of teachers were in school but were either not in the classroom or not engaged in any teaching activity while in the classroom (Table 2 - Panel B). Thus a total of 42% of teachers on the payroll were either absent or not teaching at the time of direct observations.^17^

Finally, enumerators also recorded whether a teacher had been marked as present in the log-books on the day of the visit and also on the previous day, and we see in Table 2 - Panel C that going by these records would suggest a much lower teacher absence rate of 16% using the same day's records, or as low as 10.2% using the previous day's records (this was not collected in 2003).^18^ These data highlight the importance of measuring teacher absence by direct physical verification as opposed to official records on log books.

## 4 Cross-section and panel regression results

### 4.1 Correlates of teacher absence in 2010

Table 3 presents village-level cross-sectional regressions between indicators of school quality and teacher absence in 2010. Column 1 shows the mean level of each covariate in the sample, columns 2–4 present the coefficients on each indicator in individual regressions with the dependent variable being teacher absence, while columns 5–7 do so in multiple regressions that include all the variables shown in Table 1 as regressors.

We first show the regressions with no fixed effects, then with state fixed effects, and finally with district fixed effects. The comparison of results with and without state fixed effects is important for interpretation. Many indicators of school quality vary considerably across states in a manner that is likely to be correlated with other measures of governance and development as well as the history of education investments in these states. On a similar note, while primary education policy is typically made at the state level, there is often important variation across districts within a state based on historical as well as geographical factors (Banerjee and Iyer, 2005, Iyer, 2010). Thus, specifications with district fixed effects that are identified using only within-district variation are least likely to be confounded by omitted variables correlated with historical or geographical factors. However, there may still be important fixed omitted variables across villages (such as the level of interest in education in the community) that are correlated with both measured quality of schools and teachers as well as teacher absence. We therefore present the cross-sectional regressions in Table 3 for completeness and focus our discussion on the village-level panel regressions presented in Table 4. Overall, there are few robust correlations across all specifications except that schools that have been inspected recently have lower rates of absence. One important result in the correlations is that there appears to be no significant relationship between teacher salary and the probability of teacher absence. Since salary data were not collected in the 2003 survey, this variable is not included in the panel analysis below.

### 4.2 Correlates of changes in teacher absence between 2003 and 2010

The main identification challenge in the cross-sectional regressions presented in Table 3 (and in Kremer et al. (2005)) is that we cannot rule out the possibility that the results are confounded with village-level omitted variables. The use of panel data helps mitigate these concerns since our correlations are now identified using changes in village-level measures of school inputs. Table 4 (columns 4–6) presents results from the following regression:

(1) $$\Delta Abs_{i}=\beta_{0}+\beta_{1}\cdot \Delta T_{i}+\beta_{2}\cdot \Delta S_{i}+\beta_{3}\cdot \Delta M_{i}+\beta_{Z_{i}}\cdot Z_{i}+\epsilon_{i}$$

where *ΔAbs*_*i*_ is the change in the mean teacher absence rate in government schools in village *i* between 2003 and 2010, ***Δ*** *T*_*i*_ is the change in village-level means of measures of teacher attributes, ***Δ*** *S*_*i*_ is the change in village-level means of measures of school facilities, and ***Δ*** *M*_*i*_ is the change in village-level means of measures of school monitoring and supervision. *Z*_*i*_ represents different levels of fixed effects (state or district) and *ϵ*_*i*_ is the error term. Since changes in the measures of school quality included above may be correlated, we report both individual regressions with only covariate at a time (columns 1–3) as well as multiple regressions that include all of these covariates (columns 4–6).

Since Eq.(1) differences away fixed unobserved heterogeneity at the village level (and therefore at the state and district level as well), the inclusion of state and district fixed effects in the specification controls for average state and district specific *changes* over time in both the left-hand and right-hand side variables. Thus our panel results with state and district fixed effects are least likely to be confounded with time-invariant and time-variant omitted variables.^19^ However, it is also worth noting that such a specification biases us against detecting small effects. First, first-differencing leaves us with less variation in the explanatory variables, which will increase standard errors. Second, to the extent there is measurement error in the explanatory variables, first differencing would also increase the attenuation bias. This is why we focus our discussion and interpretation of the results on the ones that are robustly significant and do not treat lack of evidence of significant effects as strong evidence in favor of null effects.

Nevertheless, the results in Table 4 suggest that several plausible narratives for the reasons for teacher absence seen in the cross-sectional data reported in Kremer et al. (2005) are not supported in the panel data regressions. In particular, unlike in Kremer et al. (2005), we find no correlation between changes in school infrastructure or proximity to a paved road and teacher absence. We also find no correlation between changes in teacher professional qualifications or professional conditions (such as regularity of pay) and changes in teacher absence.^20^

We find two robust relationships in the panel regressions, where we define ‘robust’ as correlations that are significant in both individual and multiple regressions; significant in all three main specifications (no fixed effects, state fixed effects, and district fixed effects) and consistent across all specifications (we cannot reject that the estimates are the same across specifications). We discuss these two results below.

#### 4.2.1 Reductions in STR are correlated with increased teacher absence

First, villages that saw a reduction in student-teacher ratio (STR) have significantly *higher* rates of teacher absence. A 10% reduction in STR is correlated with a 0.5% increase in average teacher absence, and these estimates remain stable when we include state and district fixed effects and are unchanged when we include a full set of controls (also measured in changes).

Changes in STR reflect changes in enrollment as well as in the number of teachers, and a higher STR may affect teacher absence through both enrollment and number of teachers. First, having more students enrolled may increase the cost to teachers of being absent since there are more students (and parents) who may complain. Second, the most common outcome for students when their teacher is absent is that they are combined with other classes/grades whose teachers are present.^21^ Thus, having more teachers in the school may make it easier for teachers to be absent (since other teachers can handle their class).^22^

These correlations should not be interpreted as causal (for instance, student enrolment may decline in response to increased teacher absence), but they are consistent with a causal relationship between increased teacher hiring and increased absence of existing teachers that has been established experimentally in India (Muralidharan and Sundararaman, 2013) and other low-income countries such as Kenya (Duflo et al., 2015). Our results provide complementary evidence and greater external validity to these experimental results, and suggest that the benefits of additional teacher hiring to reduce STR may be attenuated by increased teacher absence (in contexts with weak governance of education systems).

#### 4.2.2 Increasing monitoring is correlated with reduced teacher absence

The second robust result in the panel data estimates is the strong negative correlation between improved school monitoring and teacher absence. In each of the three visits to a school, enumerators recorded the date of the most recent inspection, and we average across the three visits across all the sampled schools in the village to construct the variable “Probability of being inspected in last 3 months”, which ranges from zero (none of the schools in the village were inspected in the prior three months in any of the three visits) to one (all the schools in the village were inspected in the prior three months in all of the three visits). We find that villages where the probability of inspection in the past three months increased from zero to one had a reduction in average teacher absence of between 6.4 and 8.2 percentage points (a 27–35 percent reduction in teacher absence).^23^ While these results are based on correlations, we present several pieces of evidence consistent with a causal effect of increased school inspections on reduced teacher absence.

First, we look at the categories of stated reasons for absence (official duty, authorized leave, and unauthorized absence), and find that increases in inspection probability are correlated only with reductions in *unauthorized* teacher absence, but not with reductions in teacher absence due to either official duty or authorized leave (Table 5). Second, we examine the extent to which changes in inspection frequency can be explained by other observable factors, and find that there are no correlations between changes in inspection frequency and changes in other observable measures of school quality that are significant across our three standard specifications (Table A5). Third, we use the technique developed by Altonji et al. (2005) to show that the ratio of unobservable to observable correlates of changes in teacher absence would have to be over a factor of 10 for these results to be completely explained by omitted variables (Table A6). Given the very rich data we have on observable changes in school quality, and the fact that our estimates are unchanged even after including state and district fixed effects, this is unlikely to be the case.^24^

Finally, these results are also consistent with experimental evidence from India that finds significant reduction in teacher absence in response to improved monitoring and rewards linked to better teacher attendance (Duflo et al., 2012). This experimental study, however, was carried out in a small sample of informal schools in one district in India. Thus, our estimates using nationally-representative panel data of rural public schools across 190 districts provide complementary evidence that improved ‘top down’ administrative monitoring may have a substantial impact on reducing unauthorized teacher absence.

In contrast, there is less evidence that increases in ‘bottom up’ monitoring by the community (measured by whether the PTA had met in the past 3  months) are correlated with reductions in teacher absence (Table 4). This is consistent with the experimental results reported in Olken (2007) on the impacts of monitoring corruption in Indonesia. These results should not be interpreted as suggesting that bottom-up monitoring *cannot* be effective, since it is also likely that they reflect differences in the effective authority over teachers possessed by administrative superiors (high) versus parents (low). PTAs in India typically do not have authority to appoint or retain regular civil-service teachers, and they cannot sanction teachers for absence or non-performance (Banerjee et al., 2010).

Inspectors and administrative superiors, on the other hand, possess considerable authority over teachers. Their powers include the ability to demand explanations for absence, to issue verbal or written warnings, to make adverse entries in teachers' performance records, to recommend against a pay increment, to suspend a teacher, and in extreme cases to initiate proceedings to fire a teacher (see Ministry of Education (1964-1966) for a detailed discussion of the design of the Indian school inspection system and the powers it provides inspectors). While it is rare for teachers in India to actually get fired for absence (Kremer et al., 2005), and also true that politically-connected teachers can evade sanctions for absence (De and Dreze, 1999, Kingdon et al., 2014), the teacher service rules include several provisions that make it possible for inspectors to significantly raise the costs of teacher absence and thereby reduce it. A striking recent example of how a motivated school inspector in India was able to reduce teacher absence is provided by Anand (Feb. 19, 2016).^25^

In interpreting the result on school inspections, it is useful to consider *why* there might be variation in the frequency of inspections across villages and what this would imply for a causal interpretation. One obvious explanation is that inspectors are more likely to visit more accessible villages, but the data do not support this hypothesis since there is no correlation between changes in the remoteness index and changes in inspection rates (Table A5).

District-level interviews on school governance in India suggest two important reasons for the variation in inspection frequency. The first is staffing. Districts are broken down further into administrative blocks, and schools within blocks are organized into clusters. School supervision is typically conducted by “block education officers” and “cluster resource coordinators”. We find that a significant fraction of these posts are often unfilled. For instance, in 19% of the cases (where we have data) even the position of the “District Education Officer (DEO)”, the senior-most education official in a district, was vacant (Centre for Policy Research, 2012).^26^ Further, there is high turnover in the education administration (the average DEO had a tenure in office of just one year) creating periods when the positions are vacant during transitions. The lack of supervisory staff at the block-level is even more acute, as 32% of these positions were estimated to be vacant in 2010 (the year of our survey) even by an official government report (13th JRM Monitoring Report, 2011). Our interviews suggest that these staffing gaps at the block and cluster level are the most important source of variation in inspection frequency within districts, since blocks and clusters without supervisory staff are much less likely to get inspected.

The second source of variation in inspections is the diligence of the concerned supervisory officer. Even if all the positions of supervisory staff were filled, there would be variation in the zealousness with which these officers visited villages/schools, which might lead to some areas being inspected more often than others based on whether they were in the coverage area of a more diligent officer or not. However, since supervisors are typically assigned a coverage area of clusters or blocks that comprise many villages, variation in monitoring frequency that is driven by supervisor-level unobservable characteristics is unlikely to be correlated with other village-level characteristics that are also correlated with absence. Of course, this source of variation has implications for thinking about the likely effectiveness of hiring new supervisory staff (some of whom may be less diligent). We discuss these in Section 5.3.

### 4.3 Teacher absence and student learning outcomes

Teacher absence reduces the effective student-teacher ratio (ESTR) for any given STR. To study the relationship between changes in teacher absence between 2003 and 2010 and changes in student learning outcomes in this period we first estimate:

(2) $$\Delta TS=\beta_{1}\Delta \log(ESTR)+\beta_{z}Controls+\epsilon$$

where changes in village-level mean normalized math test scores are regressed on changes in village-level ESTR. We find that reductions in ESTR are significantly correlated with increased student test scores (Table 6 - columns 1 and 4).^27^

Reductions in ESTR can be achieved through reducing STR as well as by reducing teacher absence. Rewriting Eq. (2), we have:

(3) $$\Delta TS=\beta_{1}\Delta \log\left(\frac{STR}{1-Absence}\right)+\beta_{z}Controls+\epsilon$$

If we relax the constraint of equal coefficients on the numerator and denominator in Eq. (3), we can rewrite it as:

(4) $$\Delta TS=\beta_{1}\Delta \log(STR)-\beta_{2}\Delta \log(1-Absence)+\beta_{z}Controls+\epsilon$$

While not very precise, the estimates in Table 6 (column 5) suggest that both reductions in *log(STR)*, and reductions in *log(1-Absence)* matter equally for improved test scores. Column 6 of Table 5 shows that once *log(ESTR)* is controlled for there is no independent effect of teacher absence on learning outcomes, suggesting that the main mechanism by which teacher absence affects learning outcomes is through increasing the ESTR. The stronger relationship between teacher absence and student learning outcomes seen in columns 2 and 3 (that do not include state or district fixed effects) suggests that teacher absence is likely correlated with other measures of education governance at the state and district levels, and highlights why our preferred specifications are the ones with district fixed effects.

Our data, which are collected seven years apart and have only mean village-level test scores, are not ideal for studying the impact of teacher absence or other school characteristics on test scores (the ideal specifications would use annual panel data on student test scores matched to these characteristics and estimate value-added models of student learning). But it allows us to present suggestive evidence on the negative correlations between teacher absence and student learning outcomes that are consistent with other studies using better data that find similar results.^28^ The results in Table 6 also help illustrate that teacher absence can attenuate the benefits of reducing STR, and that reducing *effective* STR can be done both by reducing STR and by reducing teacher absence. We consider the relative cost effectiveness of these approaches in the next section.

## 5 The fiscal cost of weak governance

### 5.1 The fiscal cost of teacher absence

High levels of teacher absence translate into considerable waste of public funds since teacher salaries are the largest component of education spending in most countries, including India. One way of estimating these costs is to calculate the total salary cost paid to teachers for days of work that they were expected to attend, but do not. Note that this is not a cost that would be saved if teacher absence were to be reduced (since the full teacher salaries would be paid in either case). However, it is standard in the corruption literature to measure the cost of corruption by the amount of public expenditure that does not reach its intended goal (often referred to as ‘leakage’), and to measure the impact of interventions to reduce corruption by quantifying the reduction in leakage, even if there is no reduction in fiscal outlay (Reinikka and Svensson, 2004, May, Reinikka and Svensson, 2005, 04/05, Niehaus and Sukhtankar, 2013, Muralidharan et al., 2016).^29^ We follow a similar approach here by first quantifying the salary cost of absence as an estimate of ‘leakage’ in education spending, and then using these costs as the metric to evaluate alternate policy approaches to reducing ESTR.

Calculating the cost of teacher absence requires us to estimate and exclude the extent of legitimate absence from our calculations. As part of the institutional background work for this project, we obtained teacher policy documents from several states across India. Analysis of these documents indicates that the annual allowance for personal and sick leave is 5% on average across states. This is close to the survey estimate of 5.9%(Table 2), but we use the official data since the stated reasons may be over-reported.

Estimating the extent of legitimate absence due to ‘official duty’ (outside the school) is more difficult because there are no standard figures for the ‘expected’ level of teacher absence for official duties. Policy norms prescribe minimal disruption to teachers during the school day and stipulate that meetings and trainings be carried out on non-school days or outside school hours. Since we are not able to verify the claim that teachers were on official duty, and there is evidence that head teachers try to cover up for teacher absences by claiming that these are due to ‘official duties', our default estimate treats half of these cases as legitimate. This gives us a base case of legitimate absence of 8%(5% authorized leave, and 3% official duty). We also consider a more conservative case where the legitimate rate of absence is 10%. This 8–10% range of legitimate absence also makes sense because the fraction of teacher observations that are classified as either ‘authorized leave’ or ‘official duty’ is in this range for the five states with the lowest overall absence rates - even treating the stated reasons for absence as being fully true (tables available on request).

To estimate the cost of teacher absence, we use teacher salary data from our surveys and use administrative (DISE) data on the number of primary school teachers by state.^30^ We provide three estimates of the fiscal cost of teacher absence based on assuming the rate of legitimate teacher absence to be 8, 9, and 10 percent% respectively, and these calculations suggest that the annual fiscal cost of teacher absence is around Rs.81 –93 billion, which is around US$1.4–1.6 billion/year at 2010 exchange rates (Table 7 - Panel A).

### 5.2 Calculating the returns to better governance in education

Using the results in Table 4, we calculate the returns to a marginal increase in the probability of a school being inspected. We make the following assumptions: (a) enough supervisory staff are hired to increase the probability of a school being inspected in the past 3  months by 10 percentage points (relative to a current probability of 56%); (b) increasing inspection probability by 10 percentage points would reduce mean teacher absence across the schools in a village by 0.64 percentage points (the most conservative estimate of the correlation between increased inspection probability and reduced teacher absence from Table 4); (c) the full cost (salary and travel) of a supervisor is 2.8 times that of a teacher; (d) a supervisor works 200  days per year and can cover 2 schools per day.^31^

The results of this estimation are presented in Table 7 (Panel B) and we see that the cost of hiring enough supervisors to increase the probability of a school being inspected by 10 percentage points is Rs.448  million/year (see Table A8 for state-level calculations). However, the reduction in wasted salary from this investment in terms of reduced teacher absence amounts to Rs.4.5  billion/year, suggesting that investing in better monitoring would lead to a reduction in ‘leakage’ of teacher salaries (defined as salary payments for days when teachers do not attend work) that is around ten times greater than the cost of increasing monitoring by hiring more supervisory staff.

### 5.3 Input augmentation versus inefficiency reduction

To compare the relative cost effectiveness of hiring more teachers (input augmentation) versus hiring more supervisors to reduce teacher absence (inefficiency reduction) as a way of reducing the ESTR, we calculate the salary cost of hiring more teachers to achieve the same reduction in ESTR that we estimate would be obtained by increasing the inspection probability by 10 percentage points. We estimate this to be Rs. 5.7  billion/year (Table 7 - Panel B; Table A8 provides detailed state-level calculations), and see that increasing the probability of inspection would be 12.8 times more cost effective at reducing ESTR than doing so by hiring more teachers (on the current margin).^32^

The difference in the relative cost effectiveness of the two policy options is large enough that hiring more supervisors rather than teachers is likely to be a more cost effective way of reducing ESTR (on the current margin) even if the supervisors were to work less efficiently than assumed in these calculations. For instance, if supervisors were absent at the same rate as teachers (say 25 %), allocating marginal funds to hire an additional supervisor would still be nearly ten times more cost effective at reducing ESTR than using those funds to hire an additional teacher.^33^

## 6 Policy implications

The main caveat to using our results to recommend a universal policy of hiring more supervisors to scale up the frequency of school inspections is that our estimates are based on correlations and may not be convincing enough to warrant a universal scale up. Nevertheless, it is worth noting that both our key results - the correlation between increased monitoring and reduced teacher absence, and the correlation between lower STR and increased teacher absence - are consistent with experimental evidence from smaller-scale, which increases our confidence in their validity. Further, our estimates are based on an expansion of existing system of inspections, and use nationwide panel data (which mitigates omitted variables concerns) representing close to a billion people, and complement results from smaller-scale randomized experiments warranting them greater external validity for several reasons.

First, while our results support results from smaller randomized experiments, there is evidence that experimentally-estimated positive results of interventions that are implemented by NGOs may not be replicated when the programs are implemented by governments (Banerjee et al., 2008). Second, there is also evidence of site-selection bias where implementing partners are more likely to be willing to rigorously evaluate programs in locations where they are more likely to be successful (Allcott, 2015). Finally, even in the absence of such a bias, most experiments are conducted in very few sites, and may yield imprecise treatment effects (for inference over a larger population) in a setting where unobserved site-specific covariates may interact with the treatment (Pritchett and Sandefur, 2013).^34^

Thus, even if small-scale experiments are unbiased within sample, they may be biased and also imprecise for population-level inference. In other words, there is likely to be a trade-off between the potential omitted variable bias in our panel-data estimates on one hand, and the advantages of greater precision, “as is” implementation, and unbiased site selection on the other. We do not attempt to quantify this trade-off in this paper since we have no objective basis of doing so. However, one way of reconciling this trade-off is to conduct a substantial nationwide expansion of school inspections by hiring more staff in the context of a large experimental evaluation. From a decision-theoretic perspective, our results are strong enough to support such a policy even if there is only a 1% chance that our estimates are causal. In Appendix B, we formally show that, barring extreme priors, a policy-maker interested in lowering effective student-teacher ratio will find it cost-effective to invest in or scale-up monitoring of teachers.

## 7 Conclusions

The central and state governments in India have considerably increased spending on primary education over the past decade. We contribute towards understanding the impact of these substantial nationwide investments in primary education in India by constructing a unique nationally-representative panel data set on education quality in rural India. We find that there has been a substantial improvement in several measures of school quality including infrastructure, student-teacher ratios, and monitoring. However, teacher absence rates continue to be high, with 23.6% of teachers in public schools across rural India being absent during unannounced visits to schools.

Using village-level panel data, we find two robust correlations in the panel data that provide external validity in nationally-representative data to results established in smaller-scale experiments. First, reductions in student-teacher ratios are strongly correlated with increased teacher absence, suggesting that increased spending on hiring additional teachers was accompanied by increased inefficiency, which may limit the extent to which additional spending may improve outcomes. Second, increases in the frequency of inspections are strongly correlated with lower teacher absence, suggesting that of all the investments in improving school quality, the one that was most effective in reducing teacher absence was improved administrative monitoring of schools and teachers. We calculate that the fiscal cost of teacher absence is over $1.5 billion per year, and estimate that investing in improved governance by increasing the frequency of monitoring would be over ten times more cost effective at increasing student-teacher contact time than doing so by hiring additional teachers.

In interpreting our results, it may be useful to think of the performance of the education system (measured by the level of teacher absence) as comprising two components - ‘inputs' into the production of education that expand with income growth (such as school infrastructure, class size, and teacher salaries), and the efficiency of the use of these inputs (which would correspond to the TFP of education production). Our results show that the Indian education system has made significant progress on the former, but made less progress on the latter. They also suggest that pivoting public expenditure away from simply augmenting inputs towards policies that increase the efficiency of inputs may considerably increase the productivity of education spending, and thereby enable achievement of improved human capital outcomes at any given level of per-capita income.

One promising way of reducing inefficiency is improving school governance and achieving such a reallocation of resources would be to expand the existing system of administrative monitoring of teachers and schools by hiring more supervisory staff. Our calculations indicate that such a marginal expansion could (on the current margin) have a significant impact on reducing teacher absence, and that this would be highly cost effective in terms of reducing the fiscal cost of weak governance. More broadly, our results suggest that the returns to investing in state capacity to better monitor the implementation of social programs in low-income countries may be quite high, and that at the very least there is a strong case for expanding such programs in the context of large experimental evaluations of “as is” implementation to obtain more precise estimates of their benefits.^35^

## Figures and Tables

**Table A2:** Description of sample: Data and attrition.

	(1)	(2)	(3)	(4)	(5)	(6)	(7)	(8)	(9)	(10)	(11)	(12)
	Year 2010 sample			Reasons for attrition (Year 2010)				Reasons for attrition (Year 2010)			Reasons for attrition	
	Sampled	Included in	Attrition	No school	No public school	School(s) refused	Other reasons	Sampled	Included in	Attrition	No data for	No data for
		analysis		in village	in village	to survey			Analysis		year 2010	year 2003
**Andhra Pradesh**	87	86	1	0	0	0	1	73	70	3	1	2
**Assam**	87	83	4	1	3	0	0	77	72	5	3	2
**Bihar**	84	81	3	1	1	0	1	84	77	7	3	4
**Chattisgarh**	80	75	5	2	1	0	2	76	69	7	4	3
**Gujarat**	88	85	3	0	3	0	0	74	71	3	3	0
**Haryana**	81	80	1	0	1	0	0	75	63	12	0	12
**Himachal Pradesh**	80	59	21	16	5	0	0	60	43	17	16	1
**Jharkhand**	84	81	3	2	1	0	0	73	58	15	3	12
**Karnataka**	89	88	1	0	1	0	0	84	82	2	1	1
**Kerala**	83	65	18	0	5	12	1	43	31	12	8	4
**Madhya Pradesh**	90	88	2	0	1	0	1	81	78	3	2	1
**Maharastra**	91	83	8	1	3	0	4	80	73	7	7	0
**Orissa**	87	83	4	2	1	0	1	79	73	6	3	3
**Punjab**	82	80	2	1	1	0	0	75	71	4	2	2
**Rajasthan**	98	94	4	1	2	0	1	85	83	2	2	0
**Tamilnadu**	87	79	8	1	5	0	2	69	62	7	5	2
**Uttar Pradesh**	113	111	2	0	2	0	0	104	100	4	2	2
**Uttaranchal**	72	67	5	1	3	0	1	57	52	5	4	1
**West Bengal**	87	87	0	0	0	0	0	70	69	1	0	1
**India**	1650	1555	95	29	39	12	15	1419	1297	122	69	53

*Source*: Authors' calculations. *Notes*: The category others include: high Naxalite activity, village not reachable, schools not functional, and schools closed in all three visits. In 2003, if a village did not have any schools, surveyors went to the neighboring village. In 2010, the village was simply recorded as having no school.

**Table 1:** Changes in key variables between 2003 and 2010, village-level data.

	(1)	(2)	(3)
	Summary statistics		Difference
	Year 2003	Year 2010	(Ho: No diff)
*A. Teacher variables*			
Have bachelors degree	0.41	0.58	0.174***
Have teacher training	0.77	0.68	−0.085***
Are contract teachers	0.06	0.30	0.233***
Are paid regularly	0.49	0.78	0.285***
Recognition scheme exists	0.50	0.81	0.309***
*B. School variables*			
Student-teacher ratio (STR)	47.19	39.80	−7.388***
Mid-day meals	0.22	0.79	0.576***
Infrastructure index (0–4)	2.14	3.35	1.205***
Has drinking water	0.80	0.96	0.160***
Has toilets	0.40	0.84	0.440***
Has electricity	0.22	0.45	0.236***
Has library	0.51	0.69	0.183***
*C. Monitoring and community variables*			
Road is within 1 km	0.69	0.78	0.092***
Probability of inspection in last 3 months	0.38	0.56	0.176***
Probability of inspection in last 2 months	0.31	0.50	0.189***
Probability of inspection in last 1 month	0.22	0.38	0.155***
Probability of PTA meeting in last 3 months	0.30	0.45	0.153***
Mean parental education (1–7 scale)	2.03	2.43	0.394***
State per-capita GDP (thousands of Rs.)	14.74	30.21	15.473***
*D. Absence variables*			
Teacher absence rate (%)	26.29	23.64	−2.64***
Effective student-teacher ratio (ESTR)	64.02	52.13	−11.89†

*Source:*Authors' calculations; Central Statistical Organization, India. *Notes*: Summary statistics (except Student-teacher ratio) are weighted by rural population of Socio-Cultural Regions (SCRs) in Census 2001. Student-teacher ratio is weighted by SCR school enrolment. Data for number of days since inspection and truncated at 99th percentile. State per-capita GDP figures are in 2004–2005 prices. Absence figures for 2003 differ slightly from the figures in the Kremer et al. (2005) paper. This is because the urban schools are removed from the sample.

† We do not conduct inference on the changes in “Effective Student-Teacher Ratio” because the data on total number of teachers are obtained from administrative (DISE) data. *** significant at 1%, ** significant at 5%, * significant at 10%.

**Table 2:** Teacher activity and reasons for absence (%).

	(1)	(2)
	Year 2003	Year 2010
*A. Physical verification: Absent*	26.29	23.64
School closed	6.08	6.60
Official teaching related duties (trainings, meetings, etc.)	5.93	5.21
Official non-teaching duties (education, health campaigns, etc.)	0.95	0.93
Official other duties (panchayat meetings, political meetings, etc.)	0.31	0.29
Authorized leave	7.62	5.91
No reason	5.40	4.70
*B. Physical verification: Present*	73.71	76.36
In classroom, actively teaching	42.93	53.08
In classroom, passively teaching	5.56	4.16
In classroom, not teaching	15.88	8.96
Found outside classroom	9.35	10.15
*C. Logbook records*		
Present today	80.93	84.06
Present last working day		89.76

*Source:* Authors' calculations. *Notes:* All figures are weighted by SCR's rural population. In 0.37% of cases, respondents said that a log-book was not maintained in the school, 0.23% refused to show log-book. In the year 2003, logbook records for previous working day were not collected. The full list of activities under for not teaching are - doing administrative/paper work, talking to/accompanying the surveyor, chatting/talking (with teachers, others), reading magazines/newspapers, sleeping, watching TV/listening to radio, doing other personal work, idle. Reasons for school closed are - opening hours but no one has arrived yet, opening hours but everyone left, and no reason.

**Table 3:** Cross-section OLS regressions results, village level, 2010 data (dependent variable: teacher absence rate (%)).

	(1)	(2)	(3)	(4)	(5)	(6)	(7)
	Summary statistics	Individual regressions			Multiple regressions		
	Year 2010	No fixed effects	w/ State fixed effects	w/ District fixed effects	No fixed effects	w/ State fixed effects	w/ District fixed effects
*Teacher variables*							
Have bachelors degree	0.58	−1.03	−6.20***	−7.51***	−1.96	−5.78**	−6.84***
	(0.32)	(1.94)	(2.39)	(2.57)	(1.76)	(2.45)	(2.59)
Have teacher training	0.68	−11.95***	−3.48	−2.92	−2.39	−2.43	−2.09
	(0.31)	(2.38)	(2.39)	(2.73)	(2.81)	(2.69)	(2.87)
Are contract teachers	0.30	10.97***	0.46	−1.12	−2.25	−0.27	−2.32
	(0.30)	(2.37)	(2.48)	(2.97)	(2.83)	(2.71)	(3.21)
Are paid regularly	0.78	−7.72***	−1.51	−1.24	−2.53	−1.10	−0.60
	(0.39)	(1.95)	(1.92)	(2.20)	(2.00)	(1.95)	(2.17)
Recognition scheme exists	0.81	−6.53***	−1.43	−1.72	−2.25	−0.19	−0.94
	(0.37)	(2.12)	(1.86)	(2.07)	(2.08)	(1.81)	(2.01)
Log of salary	9.25	−3.70***	−0.58	−0.30	0.43	−0.18	−0.15
	(0.62)	(1.08)	(0.88)	(0.96)	(1.01)	(0.94)	(0.99)
*School variables*							
Log student-teacher ratio	3.50	1.88	−2.31**	−4.07***	−2.42**	−1.65*	−3.29***
	(0.59)	(1.26)	(1.15)	(1.40)	(1.10)	(0.99)	(1.24)
Mid-day meals	0.79	0.77	0.57	2.62	0.49	0.47	2.01
	(0.38)	(1.74)	(1.80)	(2.07)	(1.70)	(1.77)	(2.03)
Infrastructure index (0–4)	3.35	−3.44***	−0.23	−0.31	−0.89	0.07	0.07
	(1.30)	(0.56)	(0.70)	(0.80)	(0.68)	(0.69)	(0.77)
Remoteness index (normalized)	0.04	0.26	0.58	0.76	0.19	0.17	0.14
	(0.95)	(0.68)	(0.59)	(0.64)	(0.64)	(0.61)	(0.65)
*Monitoring and community variables*							
Probability of inspection in last 3 months	0.56	-10.47***	−7.87***	−7.63***	−6.64***	−6.32***	−6.20***
	(0.29)	(2.07)	(2.08)	(2.39)	(1.90)	(2.04)	(2.37)
Probability of PTA meeting in last 3 months	0.45	−6.72***	−2.80**	-3.22**	−2.59*	−1.77	−2.13
	(0.48)	(1.51)	(1.17)	(1.32)	(1.33)	(1.13)	(1.32)
Mean parental education (1–7 scale)	2.43	−3.16***	0.37	−0.46	−0.90	0.64	−0.82
	(0.74)	(1.00)	(0.97)	(1.08)	(1.00)	(0.95)	(1.07)
Log state per-capita GDP	3.29	−11.01***			−9.27***		
	(0.49)	(1.51)			(2.50)		
*Regression statistics*							
Constant					74.58***		
					(11.76)		
R-squared					0.139	0.231	0.394
Adjusted R-squared					0.126	0.211	0.273
F-statistic (Inspected = PTA met)					3.186*	3.450*	2.024
Number of villages					1,555	1,555	1,555

*Source*: Authors' calculations. *Notes*: In summary statistics, standard deviations are in parentheses; in bivariate and multiple regressions, robust standard errors clustered at the district-level are in parentheses. In individual regressions (Columns 2–4), each cell is a separate regression of the row variables with the dependent variable being the change in teacher absence rate in percentage points at the village-level. In multiple regressions (Columns 5–7), each column is a single regression on all row variables. Infrastructure index variable uses availability of four items (drinking water, toilets, electricity, and library) with higher values representing better infrastructure; similarly remoteness index uses distances to nine sets of facilities, with higher values representing more remote villages. Summary statistics and regressions are weighted by SCR's population. *** Significant at 1%, ** significant at 5%, * significant at 10%.

**Table 4:** Panel OLS regression results, village-level (dependent variable: percentage points change in teacher absence).

	(1)	(2)	(3)	(4)	(5)	(6)
	Individual regressions			Multiple regressions		
	No fixed effects	w/ State fixed effects	w/ District fixed effects	No fixed effects	w/ State fixed effects	w/ District fixed effects
*Changes in teacher variables*						
Have bachelors degree	−0.42	−1.69	−3.69	−1.68	−2.31	−4.71
	(2.55)	(2.52)	(2.91)	(2.51)	(2.57)	(3.04)
Have teacher training	1.10	1.12	0.52	1.08	0.79	1.53
	(2.51)	(2.76)	(3.12)	(2.81)	(2.85)	(3.19)
Are contract teachers	−4.89	−3.39	−0.86	−5.26	−3.84	−0.83
	(3.20)	(3.41)	(3.52)	(3.37)	(3.60)	(4.03)
Are paid regularly	−0.18	−0.83	−1.47	−0.28	−0.97	−0.56
	(1.70)	(1.81)	(2.11)	(1.67)	(1.77)	(2.24)
Recognition scheme exists	−3.87**	−3.34*	−3.69**	−3.06*	−2.03	−3.34
	(1.76)	(1.75)	(1.87)	(1.71)	(1.69)	(2.23)
*Changes in school variables*						
Log student-teacher ratio	−5.33***	−4.89***	−4.48**	−5.56***	−4.95***	−4.69***
	(1.83)	(1.68)	(1.91)	(1.81)	(1.57)	(1.78)
Mid-day meals	1.31	1.81	4.19	1.62	0.95	2.14
	(1.73)	(2.09)	(2.59)	(1.73)	(2.08)	(2.85)
Infrastructure index (0–4)	−1.10*	−0.97	−1.01	−0.97	−0.68	−0.96
	(0.66)	(0.69)	(0.76)	(0.66)	(0.66)	(0.78)
Remoteness index (normalized)	−1.16	−0.93	−0.55	−1.25	−1.04	−0.81
	(1.05)	(1.06)	(1.08)	(1.00)	(0.95)	(1.13)
*Changes in monitoring and community variables*						
Probability of inspection in last 3 months	−8.23***	−7.31***	−6.60***	−7.35***	−6.56***	−6.41***
	(1.94)	(1.98)	(1.91)	(1.83)	(1.83)	(2.01)
Probability of PTA meeting in last 3 months	−1.65	−3.18*	−3.80**	-1.71	−2.08	−2.96
	(1.74)	(1.63)	(1.72)	(1.67)	(1.64)	(2.02)
Mean parental education (1–7 scale)	−1.29	−0.09	0.48	−1.13	−0.46	0.51
	(1.40)	(1.38)	(1.44)	(1.29)	(1.32)	(1.46)
Log state per-capita GDP	−4.69			−6.18		
	(7.39)			(7.18)		
*Regression statistics*						
Constant				3.43		
				(5.50)		
R-squared				0.071	0.143	0.346
Adjusted R-squared				0.054	0.115	0.188
F-statistic (Inspected = PTA met)				4.419**	2.921*	1.268
Number of villages				1,297	1,297	1,297

*Source:* Authors' calculations. *Notes:* In summary statistics, standard deviations are in parentheses; in bivariate and multiple regressions, robust standard errors clustered at the district-level are in parentheses. In individual regressions (Columns 1–3), each cell is a separate regression of the row variables with the dependent variable being the change in teacher absence rate in percentage points at the village-level. In multiple regressions (Columns 4–6), each column is a single regression on all row variables. Infrastructure index variable uses availability of four items (drinking water, toilets, electricity, and library) with higher values representing better infrastructure; similarly remoteness index uses distances to nine sets of facilities, with higher values representing more remote villages. Regressions are weighted by SCR's population. *** Significant at 1%, ** significant at 5%, * significant at 10%.

**Table 5:** Correlation between inspection frequency and teacher absence by reason (panel analysis: year 2003 and year 2010 data).

	(1)	(2)	(3)	(4)	(5)	(6)
	Individual regressions			Multiple regressions		
	No fixed effects	w/ State fixed effects	w/ District fixed effects	No fixed effects	w/ State fixed effects	w/ District fixed effects
*Panel A: Change in teacher absence due to official duty*						
Change in probability of inspection	−1.77*	−1.05	−1.45	−1.43	−1.00	−1.49
	(0.92)	(0.85)	(0.97)	(0.91)	(0.83)	(0.96)
*Panel B: Change in teacher absence due to authorized leave*						
Change in probability of inspection	0.77	0.42	0.59	0.59	0.33	0.50
	(0.83)	(0.84)	(0.91)	(0.85)	(0.84)	(0.91)
*Panel C: Change in teacher absence due to unauthorized leave*						
Change in probability of inspection	−7.22***	−6.68***	−5.74***	−6.51***	−6.07***	−5.41***
	(1.69)	(1.86)	(1.78)	(1.66)	(1.79)	(1.75)

*Source*: Authors' calculations. *Notes*: Robust standard errors clustered at the district-level are in parenthesis. Regressions are weighted by SCR's population. Multiple regressions include full set of controls as Table 3, coefficients not shown for brevity. *** Significant at 1%, ** significant at 5%, * significant at 10%.

**Table 6:** Panel OLS regression results, village-level (dependent variable: change in normalized math score).

	(1)	(2)	(3)	(4)	(5)	(6)
	Multiple regressions			Multiple regressions		
	No fixed effects			District fixed effects		
Change in Log ESTR	−0.199***		−0.097	−0.147**		−0.142*
	(0.069)		(0.083)	(0.071)		(0.082)
Change in log STR		−0.100			−0.149*	
		(0.083)			(0.083)	
Change in log (1-absence)		0.369***			0.127	
		(0.106)			(0.115)	
Change in absence rate			−0.005**			−0.000
			(0.002)			(0.002)
Controls	yes	yes	yes	yes	yes	yes
F-statistic and p-value:		4.35			0.03	
dlogSTR = -dlog(1-Absence)		(0.0381)			(0.8707)	
R-squared	0.053	0.058	0.060	0.432	0.433	0.432
Number of villages	1149	1150	1149	1149	1150	1149

*Source:* Authors' calculations. *Notes:* Robust standard errors clustered at the district level are in parenthesis. All regressions are weighted by SCR population. Regressions include the full set of controls as Table 3, coefficients not shown for brevity. *** Significant at 1%, ** Significant at 5%, * Significant at 10%.

**Table 7:** The fiscal cost of absence (year 2010).

	(1)
*Panel A: Fiscal cost of absence*	
Average monthly salary (Rs).	11,368
Number of teachers	3,949,338
Total loss due to absence (Rs. millions)	
Allowed absence (8%)	92,699
Allowed absence (9%)	86,773
Allowed absence (10%)	80,847
*Panel B: Marginal returns to investing in governance*	
Student teacher ratio (STR)	31.7
Effective student teacher ratio (ESTR)	41.5
Effect of increase inspection probability by 10 percentage points	
Annual cost (Rs. millions)	448.0
Annual savings from reduced teacher absence (Rs. millions)	4509.6
Expected effective student teacher ratio	41.1
Cost to produce equal effect through teacher hiring	5742.0

*Source:* Authors' calculations; DISE. *Notes:* All figures are in 2010 prices. Teacher salaries data are from Teacher Long and School Census Data. Data on number of teachers, number of schools, and enrollment are from DISE State Report Cards. Simulation assumes that one inspection every 3 months reduces absence linearly by 6.4 percentage points. Inspector costs are assumed to be two times teacher salaries, travel costs are assumed to be 80% of monthly salary, and an inspector is assumed to work 200 days a year and inspect two schools every day. Detailed calculations are available in appendix tables A9 and A10.

**Table A1:** Description of sample: Panel construction.

	(1)	(2)	(3)	(4)	(5)	(6)	(7)	(8)	(9)
	Number of villages				Reasons for reduction in panel size				
	Year 2003	Year 2010	Panel	**Reduction in**	More than 8 panel	Village population	Village population	Village not found	Other reasons
				**panel size**	villages in district	less than 250	more than 10,000	in Census 2001	
**Andhra Pradesh**	81	87	73	8	3	0	4	1	0
**Assam**	98	87	77	21	5	3	0	10	3
**Bihar**	94	84	84	10	10	0	0	0	0
**Chattisgarh**	85	80	76	9	1	0	1	2	5
**Gujarat**	82	88	74	8	2	2	2	0	2
**Haryana**	81	81	75	6	3	1	1	1	0
**Himachal Pradesh**	89	80	60	29	2	22	0	4	1
**Jharkhand**	87	84	73	14	7	4	0	1	2
**Karnataka**	91	89	84	7	2	3	2	0	0
**Kerala**	83	83	43	40	0	0	40	0	0
**Madhya Pradesh**	88	90	81	7	3	1	2	1	0
**Maharastra**	85	91	80	5	2	0	3	0	0
**Orissa**	92	87	79	13	4	5	1	3	0
**Punjab**	78	82	75	3	0	0	1	2	0
**Rajasthan**	91	98	85	6	1	1	0	4	0
**Tamilnadu**	84	87	69	15	5	0	6	4	0
**Uttar Pradesh**	114	113	104	10	9	1	0	0	0
**Uttaranchal**	80	72	57	23	6	14	1	2	0
**West Bengal**	85	87	70	15	4	3	5	1	2
**India**	1,668	1,650	1,419	249	69	60	69	36	15

*Source*: Authors' calculations. *Notes*: The upper population cutoff for all states was 10,000 as per the 1991 census, except Kerala where the cutoff was 20,000. The category others include: replaced because high Naxalite activity (6 villages), replaced because duplicate in 2003 sample (2 villages), replaced because district was replaced (2 villages) replaced because village too remote (1 village), replaced because name missing in 2003 list (1 village), replaced because of floods in village (2 village), replaced because village could not be located (1 village).

**Table A3:** Description of sample: Final sample.

	(1)	(2)	(3)	(4)	(5)	(6)	(7)	(8)
	Year 2010 sample			Panel				
	Number of	Number of	Number of	Number of	Number of	Number of	Number of	Number of
	villages	schools	teachers	villages	schools in 2003	schools 2010	in 2003	teachers in 2010
**Andhra Pradesh**	86	130	509	70	107	107	372	405
**Assam**	83	150	525	72	122	134	437	473
**Bihar**	81	124	757	77	112	119	341	731
**Chattisgarh**	75	100	450	69	94	92	259	412
**Gujarat**	85	119	944	71	101	98	419	798
**Haryana**	80	105	520	63	85	83	386	395
**Himachal Pradesh**	59	70	270	43	44	51	172	205
**Jharkhand**	81	132	493	58	76	94	244	374
**Karnataka**	88	120	572	82	117	112	598	530
**Kerala**	65	105	608	31	57	50	353	307
**Madhya Pradesh**	88	146	476	78	116	133	367	427
**Maharastra**	83	98	495	73	96	88	441	451
**Orissa**	83	114	483	73	88	101	295	439
**Punjab**	80	88	469	71	75	76	355	417
**Rajasthan**	94	141	671	83	132	121	497	565
**Tamilnadu**	79	96	445	62	124	75	455	363
**Uttar Pradesh**	111	135	616	100	131	119	442	542
**Uttaranchal**	67	73	207	52	61	57	177	151
**West Bengal**	87	151	668	69	108	121	331	531
**India**	1555	2197	10,178	1297	1846	1831	6941	8516

*Source*: Authors' calculations.

**Table A4:** Absence rate of teachers & student-teacher ratios in rural public schools by state by year.

	(1)	(2)	(3)	(4)	(5)	(6)	(7)	(8)	(9)
	Absence rates(%)			Student- teacher ratio			Effective student-teacher ratio		
	Year 2003	Year 2010	Change	Year 2003	Year 2010	Change	Year 2003	Year 2010	Change†
**Andhra Pradesh**	23.38	21.48	−1.90	27.51	25.79	−1.71	35.90	32.85	−3.05
**Assam**	36.15	26.26	−9.89***	28.21	36.07	7.86***	44.18	48.92	4.74
**Bihar**	39.42	28.69	−10.73***	72.44	69.01	−3.43	119.57	96.78	−22.79
**Chattisgarh**	30.47	14.20	−16.28***	42.12	33.05	−9.07***	60.59	38.52	−22.07
**Gujarat**	17.92	16.14	−1.77*	40.42	31.94	−8.48***	49.24	38.09	−11.15
**Haryana**	21.07	17.75	−3.31**	34.40	36.34	1.94	43.58	44.18	0.60
**Himachal Pradesh**	22.67	30.74	8.07***	18.04	21.73	3.69**	23.33	31.38	8.04
**Jharkhand**	43.50	45.84	2.34	52.30	42.84	−9.47***	92.57	79.09	−13.48
**Karnataka**	22.60	23.93	1.33	29.07	23.62	−5.45***	37.56	31.05	−6.51
**Kerala**	19.60	15.79	−3.81***	24.84	24.49	−0.36	30.90	29.08	−1.82
**Madhya Pradesh**	18.19	26.34	8.16***	37.19	46.57	9.39***	45.45	63.23	17.78
**Maharastra**	15.43	14.12	−1.31	34.54	28.66	−5.88***	40.84	33.38	−7.47
**Orissa**	21.69	14.24	−7.46***	47.01	36.63	−10.38***	60.04	42.72	−17.32
**Punjab**	36.66	13.54	−23.13***	30.80	31.43	0.63	48.63	36.36	−12.28
**Rajasthan**	25.13	22.72	−2.42*	38.91	32.05	−6.86***	51.97	41.47	−10.50
**Tamilnadu**	20.43	12.92	−7.51***	29.56	25.85	−3.71**	37.15	29.69	−7.47
**Uttar Pradesh**	26.72	31.21	4.49***	69.37	47.40	−21.97***	94.66	68.90	−25.76
**Uttaranchal**	32.29	21.02	−11.27***	24.49	31.02	6.54**	36.17	39.28	3.12
**West Bengal**	26.41	20.97	−5.44***	58.23	41.61	−16.62***	79.12	52.65	−26.47
**India**	**26.29**	**23.64**	−2.64***	**47.19**	**39.80**	−7.39***	**64.02**	**52.13**	**−11.89**

*Source*: Authors' calculations; DISE *Notes*: All figures are weighted by SCR's rural population. Absence figures for 2003 differ from the figures in the Kremer et al. (2005) paper. This is because the urban schools are removed from the sample. We do not conduct inference on the changes in “Effective student-teacher ratio” because the data on total number of teachers are obtained from administrative (DISE) data.

†We do not conduct inference on the changes in “Effective student-teacher ratio” because the data on total number of teachers are obtained from administrative (DISE) data. *** Significant at 1%, ** significant at 5%, * significant at 10%.

**Table A5:** Panel OLS regression results, village-level(dependent variable: Change in probability of inspection in past 3months).

	(1)	(2)	(3)	(4)	(5)	(6)
	Individual regressions			Multiple regressions		
	No fixed	w/state	w/district	No fixed	w/state	w/district
	effects	fixed effects	fixed effects	effects	fixed effects	fixed effects
*Changes in teacher variables*						
Have bachelors degree	−0.003	0.042	0.039	0.006	0.037	0.030
	(0.046)	(0.053)	(0.050)	(0.046)	(0.051)	(0.055)
Have teacher training	0.041	0.054	0.085	0.029	0.046	0.064
	(0.056)	(0.057)	(0.054)	(0.053)	(0.055)	(0.061)
Are contract teachers	0.055	0.063	−0.040	0.108*	0.088	−0.009
	(0.053)	(0.073)	(0.069)	(0.059)	(0.070)	(0.082)
Are paid regularly	−0.036	−0.010	−0.010	−0.037	−0.005	−0.004
	(0.030)	(0.035)	(0.035)	(0.031)	(0.035)	(0.041)
Recognition scheme exists	0.069**	0.062**	0.020	0.067**	0.060*	0.023
	(0.028)	(0.031)	(0.032)	(0.028)	(0.031)	(0.037)
*Changes in school variables*						
Log student-teacher ratio	0.055*	0.032	0.029	0.049	0.024	0.012
	(0.031)	(0.032)	(0.034)	(0.030)	(0.031)	(0.037)
Mid-day meals	0.007	−0.008	−0.024	0.018	−0.008	−0.017
	(0.032)	(0.041)	(0.046)	(0.034)	(0.042)	(0.050)
Infrastructure index (0-4)	0.010	0.011	0.005	0.006	0.011	0.004
	(0.012)	(0.013)	(0.015)	(0.013)	(0.013)	(0.015)
Remoteness index (normalized)	−0.023	−0.026	−0.032	−0.024	−0.024	-0.028
	(0.022)	(0.022)	(0.020)	(0.021)	(0.021)	(0.024)
*Changes in monitoring and community variables*						
Probability of PTA meeting in last 3 months	0.018	0.052**	0.068**	0.033	0.053**	0.070**
	(0.023)	(0.024)	(0.029)	(0.023)	(0.024)	(0.027)
Mean parental education (1–7 scale)	−0.03	−0.04	−0.04**	−0.04	−0.04*	−0.05**
	(0.026)	(0.026)	(0.022)	(0.023)	(0.024)	(0.025)
Log state per-capita GDP	−4.69			0.40**		
	(7.392)			(0.167)		
*Regression statistics*						
Constant				−0.13		
				(0.138)		
R-squared				0.051	0.093	0.315
Adjusted R-squared				0.034	0.065	0.152
Number of villages				1300	1300	1300

*Source*: Authors' calculations. *Notes*: Robust standard errors clustered at the district-level are in parentheses. Infrastructure index variable uses availability of four items (drinking water, toilets, electricity, and library) with higher values representing better infrastructure; similarly remoteness index uses distances to nine sets of facilities, with higher values representing more remote villages. Regressions are weighted by SCR's population. *** Significant at 1%, ** significant at 5%, * significant at 10%.

**Table A6:** Selection on observables and selection on unobservables (An application of Altonji, Elder and Taber (2005)).

	(1)	(2)	(3)
	Dependent variable: Percentage change in absence		
	Treatment variable: Increase in inspection probability		
Coefficient on treatment	Unconstrained coefficient	Estimate of bias	Implied ratio [(1)/(2)]
Base specification (no fixed effects)	−5.560***	−2.298	2.598
	(1.551)		
State fixed effects	−5.343***	−0.856	6.176
	(1.499)		
District fixed effects	−5.118***	−0.502	10.189
	(1.765)		

*Source:* Authors' calculations. *Notes:* Robust standard errors clustered at the district-level are in parenthesis. Regressions include full set of controls as Table 3, coefficients not shown for brevity. We discretize the main variable of interest - Change in probability of inspection. Villages where inspection rates increased between 2003 and 2010 are coded as 1, and 0 otherwise. 52% of villages experienced an increase in inspection, and inspection rates fell or did not change in the remaining 48%. *** Significant at 1%, ** significant at 5%, * significant at 10%.

**Table A7:** The fiscal cost of absence (year 2010).

	(1)	(2)	(3)	(4)	(5)
	Average monthly	Number of	Total loss due to absence (millions of Rs.)		
	teacher salary (Rs.)	teachers	Allowed absence:	Allowed absence:	Allowed absence:
			8%	9%	10%
**Andhra Pradesh**	10,299	347,875	6374	5901	5428
**Assam**	9567	167,161	3855	3644	3433
**Bihar**	8645	336,359	7942	7559	7175
**Chattisgarh**	8290	155,573	1055	885	715
**Gujarat**	15,804	198,584	3374	2960	2546
**Haryana**	16,236	77,980	1630	1463	1296
**Himachal Pradesh**	12,199	48,507	1776	1698	1620
**Jharkhand**	9734	135,690	6598	6423	6249
**Karnataka**	10,897	195,929	4489	4207	3925
**Kerala**	10,751	54,976	608	529	451
**Madhya Pradesh**	9294	267,846	6027	5698	5370
**Maharastra**	17,246	288,914	4025	3367	2710
**Orissa**	9382	192,119	1484	1246	1008
**Punjab**	12,654	105,930	980	803	626
**Rajasthan**	14,165	271,205	7463	6956	6448
**Tamilnadu**	18,489	150,820	1811	1443	1075
**Uttar Pradesh**	10,370	491,455	15,615	14,942	14,269
**Uttaranchal**	17,155	45,782	1350	1246	1143
**West Bengal**	10,555	416,633	7527	6946	6366
**India**	11,368	3,949,338	92,699	86,773	80,847

*Source*: Authors' calculations; DISE. *Notes*: 2010 teacher salaries are from Teacher Long and School Census Data. Data on total number of teachers are from DISE State Report Cards. All figures are in 2010 prices.

**Table A8:** Marginal returns to investing in governance.

	(1)	(2)	(3)	(4)	(5)	(6)
	Student-teacher ratio (2009–2010)		Effect of increasing probability of inspection in past 3 months by 10 percentage points			Cost to produce equal effect through teacher hiring
	Student-teacher ratio	Effective student-teacher ratio	Annual cost (Rs. millions)	Annual savings from reduced teacher absence (Rs. millions)	Expected effective student-teacher ratio	Annual cost (Rs. millions)
**Andhra Pradesh**	17.8	22.7	31.0	350.8	22.5	433.5
**Assam**	24.5	33.2	15.9	154.5	33.0	204.2
**Bihar**	58.2	81.6	21.2	273.6	80.8	374.9
**Chattisgarh**	24.5	28.5	13.9	120.1	28.3	135.0
**Gujarat**	29.8	35.5	19.1	291.8	35.3	336.2
**Haryana**	26.8	32.5	8.8	118.9	32.3	139.8
**Himachal Pradesh**	15.4	22.2	6.8	56.0	22.0	79.2
**Jharkhand**	41.3	76.2	14.8	127.9	75.3	236.3
**Karnataka**	23.6	31.0	18.5	201.6	30.8	257.7
**Kerala**	19.6	23.2	2.0	56.3	23.1	64.5
**Madhya Pradesh**	39.8	54.0	40.6	250.9	53.5	332.1
**Maharastra**	25.7	29.9	45.0	486.8	29.7	546.8
**Orissa**	29.4	34.3	20.5	177.5	34.1	199.7
**Punjab**	20.5	23.7	10.2	137.4	23.5	153.2
**Rajasthan**	26.2	33.9	40.0	361.6	33.6	454.5
**Tamilnadu**	28.3	32.5	24.6	264.9	32.3	293.2
**Uttar Pradesh**	40.1	58.2	58.4	489.4	57.7	697.1
**Uttaranchal**	20.6	26.0	10.7	73.3	25.8	90.0
**West Bengal**	32.3	40.8	30.1	409.4	40.5	502.5
**India**	31.7	41.5	448.0	4509.6	41.1	5742.0

*Source*: Authors' calculations; DISE. *Notes*: Number of schools, number of teachers, and enrollment figures are from administrative (DISE) data. Simulation assumes that one inspection every 3 months reduces absence linearly by 6.4 percentage points. Inspector costs are assumed to be two times teacher salaries, travel costs are assumed to be 80% of monthly salary, and an inspector is assumed to work 200 days a year and inspect two schools every day.

## Appendix A Sampling and construction of village-level panel dataset

The original survey in 2003 covered the 19 largest states of India by population (except Delhi). Within each state, 10 districts were sampled using Probability Proportional to Size (PPS) and within each district, 10 primary sampling units (PSUs, which could be villages or towns) were sampled by PPS, thereby yielding a nationally representative sample of 1,900 PSUs across 190 districts (including towns and villages). The exception is Uttar Pradesh where 11 districts were sampled and Uttaranchal where 9 districts were sampled (since Uttaranchal had only 9 districts, and Uttar Pradesh is the largest state in India). Additionally, to account for the considerable geographic diversity within Indian states, the sample was stratified by geographic socio-cultural region (SCRs), and the 10 districts in each state were allocated to SCRs proportional to the population of the SCRs. Similarly, the 10 PSUs within each district were allocated to villages/towns proportional to the rural/urban population split in the district. All sampling was done on the basis of the 1991 census, since that was the latest Census data available at the time of the study.

The 2003 sample was augmented to include 241 villages from the REDS survey (Foster and Rosenzweig, 1996). Since the REDS villages are drawn as a representative sample within districts, including these villages does not change the representativeness of the sample. If a REDS district was in our main sample, the REDS villages were included (typically 2 to 4 per REDS district) and additional villages were sampled randomly to make up the total desired sample size. If a REDS district was not in our sample, those villages were covered in addition to our core sample. Including these villages provides more precise estimates of outcomes in the SCRs where they are located. All analysis is weighted by SCR populations, so the final estimates continue to be nationally-representative on a population weighted basis.

The final sample in 2003 comprised of 2141 rural and urban PSUs across 19 states of India. In 2010, since the survey only covered rural areas, the sample size was reduced from 10 to 8 villages per district. All districts in the 2003 sample were retained in the 2010 study, with three exceptions where full-urban districts sampled in 2003 were replaced with a new PPS sampled district from the same SCR. The three replaced districts are Hyderabad in Andhra Pradesh, Ahmedabad in Gujarat, and Greater Bombay in Maharashtra, which are highly urban districts containing their respective state capitals.

As we highlight in the paper, to meet our objective to maintain both representativeness of the current landscape of schools in rural India and to maximize the size of the panel, we retain villages from the 2003 study to the extent possible. In Column 1 of Table A1, we provide state-wise counts of rural PSUs that were sampled in the 2003 study. After removing PSUs in the three replaced districts altogether and all other urban PSUs from the 2003 study, the maximum panel size we could draw, including the REDS villages was 1,668. We sampled a 2003 village by default as long as the village had a population between 250 and 10,000 as per the 1991 Census, and we could locate the village in the 2001 Census.^36^ In districts where we had more than 8 rural PSUs in 2003, we sampled 8 PSUs randomly. The lower cutoff on population was based on the Government of India's mandate that all rural habitations exceeding 250 people should have a school with 1 km. Since villages and hamlets can be absorbed into expanding cities over time, we match the originally sampled 1991 village to the villages in the 2001 Census to make sure that the sampled village still exists.

From the 2003 list of 1668 villages, we had to remove 249 from the 2010 sampling frame for reasons we discuss below (see Columns 5 through 9 of Table A1 for the distribution of these villages across states). 69 villages were dropped because they fall in districts that had more than 8 villages in the 2003 round. A further 129 villages were removed either because their population was below 250, or had far exceeded 10,000 in the 2001 Census (20,000 for Kerala). A total of 36 villages could not be located in the 2001 Census (suggesting that they had either been depopulated or absorbed into nearby towns). Finally, 15 villages were replaced due to safety, logistical and accessibility reasons. Thus, our sample consists of 1419 villages from 2003 (Table A1 - column 3).

In districts where we had fewer than 8 villages in the 2003 sample (recall that the rural/urban sampling within districts was done on the basis of population ratios, and thus districts where over 25% of the population in 1991 was urban would have fewer than 8 villages), we sample more villages as required to reach a minimum sample size of 8 villages per district for the 2010 survey. The new villages were sampled PPS from the universe of eligible villages in the 2001 Census that were not already sampled. The cross-section sample (including REDS villages) thus consists of 1,650 villages (Table A1 - column 2).

Of the 1650 villages that comprise our 2010 sample, data from 1555 villages were included in the analysis presented in this paper (Table A2 - column 2). First, we found that 29 of the 1650 villages have no schools in the village. A large proportion of these villages (12 out of 29) are in Himachal Pradesh, which is a sparsely populated mountainous state, with many small habitations. Another 39 villages did not have a public school within the village, but did have a private school. Since this paper focuses on changes in public schools, these villages are not included in the analysis. In Kerala, we lose another 12 villages, because all schools in the village refused to be allowed to be surveyed.^37^ Finally, we drop 15 more villages from our analysis because in these villages, schools were either not functional or closed in all three visits, which means we were unable to complete surveys. A state-level breakdown of these 95 villages is provided in Columns 4–7 of Table A2. The decline in the cross-section sample size for reasons we discussed above, also reduces the number of villages for which we have panel data. After accounting for the above 95 villages and 53 villages in 2003 for which we have no data (for similar reasons as outlined for the 2010 survey round), our final panel size is 1297 villages. These 1297 villages form the core of our analysis.

To ensure a representative sample of schools, enumerators first conducted a full mapping of all public and private schools in each sampled village. Enumerators conducted “Participatory Resource Assessments” with households at multiple locations (at least three) within each village to obtain a list of all primary schools within the boundary of the village. All enumerated schools were administered a short survey that included questions on school administration such as management (public or private), enrollment, infrastructure etc. Enumerators also collected a list of all teachers in the school and their demographic characteristics. This school listing in each sampled village provided the frame for school sampling. We sampled up to three schools per village. If the village had three or fewer schools, all schools were sampled. If the village had more than three schools, we stratified the schools by management type and randomly sampled two public schools and one private school to the extent possible. In the event that there were only one public school and two or more private schools, one government and two private schools were sampled. Table A3 provides the state-level breakdown of the number of schools and teachers in the final (public school) sample used in this paper (both cross section and panel).

## Appendix B A decision-theoretic case for scale-ups of monitoring with an RCT

Formally, consider a simple binary policy regarding the number of supervisors to be hired that can take the values {0,1}, where the current policy is {0} and {1} represents a ‘new’ policy of hiring enough supervisors to ensure that all schools are inspected once in three months. The costs of the new policy are the additional salary and operational costs of hiring supervisors, and the benefits are the reduced fiscal cost of teacher absence. Denote these by *C*{1} and *B*{1} respectively, and assume that it is optimal to implement the policy if *B*{1} > *C*{1}. However, while *C*{1} is known, there is uncertainty around *B*{1} and a randomized controlled trial (RCT) in the context of a policy movement towards {1} would reduce the uncertainty around *B*{1}.

Suppose that after the trial, the likelihood that the optimal policy switches from {0} to {1} is *p* and that the expected per-period net benefit of such a switch is *q*. Let cost of data collection and analysis of a trial be *C*{*data*} and the discount rate be *r*. Let the period of the trial be one year and the fraction of the population participating in the trial be *N*. Half of those in the trial are allocated to a treatment group and the other half to a control group. Since data collection will be based on a representative sample of trial sites, we assume that *C*{*data*} does not vary with the size of the trial. The one period cost of the trial is then *C*{*data*} + (*N*/2) * *C*{1}. The benefits of the trial are the expected one-period benefit of the new policy (during the trial) and the discounted benefits of switching to a new policy (in perpetuity), weighted by the probability that the trial will lead to a switch in the policy. Thus, the trial should be conducted as long as:

(B.1) $$C\{data\}+(N/2)*C\{1\}<(N/2)*B\{1\}+\frac{pq}{r}*\frac{1}{1+r}$$

To focus on the benefits of learning if the optimal policy should be {1} instead of {0}, we abstract away from the benefit of the policy during the trial period and the one-period delay in implementing the new policy (if found to be optimal), in which case the trial should be conducted as long as:

(B.2) $$C\{data\}+(N/2)*C\{1\}<\frac{pq}{r}$$

Using our results to calibrate these quantities, it is straightforward to see that the expected benefits of a trial are very large even under extremely conservative assumptions. The estimates in Table A8 suggest that the marginal cost of {1} would be $33 million and that the marginal benefit would be $331 million (using our panel data estimates).^38^ Thus, if our estimates are true, *q* would be around $300 million/year, and using a discount rate of 10%, the net present value of moving to {1} would be $3 billion. Now suppose there is only a 1% chance that the causal impacts of inspections on teacher absence are as great as the panel data estimates presented here and that there is a 99% chance that the causal impacts of inspection are not significantly different from zero (i.e. *p* = 0.01). Even then, we see that $\left[\frac{q}{\left(1+r\right)},*,p,q\right]$ is $30 million.

On the cost side, we conservatively estimate (using data from our own field costs) that a highly-powered trial would have *C*{*data*} in the range of $1 million. A trial with an N of 0.06 would be a very large trial and could cover a nationally-representative sample across all major Indian states, but would only cost $1 million/year.^39^ Thus, even including all costs of data collection, the upper bound of the costs of such a trial would be $2 million compared to a likely lower-bound expected benefit of $30 million.^40^ An expansion of school inspections in the context of an experimental evaluation would therefore make sense even if there was only a 1% chance of the true effects being the same as our panel-data estimates.

If we use a medical ethics perspective in this setting, we also need to consider the costs of *not* providing a treatment that is known (or highly likely) to be effective. In this case, that would be the foregone one-period benefit of scaling up the treatment immediately (which we estimate to be around $300 million). Thus, depending on their prior beliefs, and the extent to which our panel data estimates shift these priors, some policy makers may choose to switch the policy regime from {0} to {1} immediately. However, the point of our exercise above is to show that policy makers, depending on their beliefs, should either implement {1} immediately or do a large expansion in the context of an RCT as described above, but it would only be under an extreme set of beliefs (that there is less than a 1% chance of our panel-data estimates being truly causal) that a policy maker would do nothing based on our results.

## Footnotes

1 In the year 2004–2005, India's education budget was Rs.1528 billion ($25 billion) and it more than doubled to Rs.3783 billion ($60 billion) in 2009–2010 (Pratham, 2010).

2 Official records were obtained from the “District Information System for Education” data (commonly known as the DISE data).

3 The all-India weighted average teacher absence estimated in 2003 was 25.2%; the corresponding figure for the rural sample was 26.3%. The panel survey only covered the rural sample.

4 These point estimates are significant and similar in both individual and multiple regressions, and in specifications with no fixed effects, with state fixed effects, and with district fixed effects. However, even with the use of panel data, we cannot rule out the possibility of time-varying omitted variables at the village-level that are correlated with village-level changes in inspections or STR. To assess the likely bias due to unobserved heterogeneity, we show using the technique developed by Altonji et al. (2005) that the ratio of unobservable to observable correlates of changes in teacher absence would have to be over 10 for our results to be completely explained by omitted variables. We argue that this is unlikely given our rich data on observable changes in school-quality (see Section 4.2.2).

5 See http://indiabudget.nic.in/budget2012-2013/ub2012-13/rec/tr.pdf.

6 The World Bank's *World Development Report 2004* provided estimates of provider absence in both health and education for a sample of low-income countries (Chaudhury et al., 2006, World Bank, 2003). These numbers have been widely cited in policy discussions, and reduction in provider absence rates is often included as an objective in aid agreements between donors and aid recipients.

7 Such misallocation in education spending is also seen in other low-income countries. An even more striking example is provided by de Ree et al. (2015) who experimentally study the intensive-margin impacts of an Indonesian policy reform that doubled teacher pay across the board (at a similar cost of $5 billion/year) and find that the teacher pay increase had no impact on student learning.

8 This is also why the 2010 wave did not include urban areas. Since school-level identifiers from the 2003 survey were not preserved (for confidentiality reasons), the panel needed to be constructed at the town/village level. However, since the fraction of urban schools covered in 2003 (relative to the total number of schools in the sampled towns) was very small, it was not possible to construct a credible panel-data estimate of school quality in towns. In rural areas, this was not a concern because we typically covered all the public schools in a village (in 84.2% of the cases) and had a mean coverage rate of 82.7% of public schools in the sampled villages.

9 While the school year is not identical across states, it typically runs from mid-June to mid-April. The three visits therefore spanned two academic years, with the first visit being made during January–March 2010, the second visit being made during June–August, and the third visit during August–October 2010.

10 This was important given the widespread possession of cell phones among teachers, which would allow them to call up absent colleagues on seeing external visitors in the school measuring teacher absence.

11 Not all interviews could be completed. Most non-responses were at the teacher as opposed to the school level (since absent teachers could not be interviewed, whereas school data could be obtained from either the head teacher or any other senior teacher). These non-responses are unlikely to affect the analysis in this paper because the panel-data analysis will focus on aggregated data at the village level as opposed to the individual data at the teacher level.

12 Field teams obtained lists of state and national school holidays in advance of creating the field plans and ensured that no visits were conducted on these days.

13 Even in the absence of school construction, the survey firm did not retain school and teacher level identifiers from the 2003 survey (complying with data protection norms), which would have made it difficult to construct a school-level panel (especially for villages with multiple schools).

14 We construct an index of school infrastructure by adding indicators for the existence of four items: drinking water, toilets, electricity, and a library. Table 1 provides summary statistics for each indicator and the overall index.

15 We find large variation in teacher absence rates across states ranging from 12.9% in Tamilnadu to 45.8% in Jharkhand. Teacher absence rates declined in 14 out of 19 states with significant reductions in 12 states, and five states having teacher absence rates below 15%. However, the ESTR in 2010 in three of India's most educationally backward states (Bihar, Jharkhand, and Uttar Pradesh) was as high as 97, 79, and 69. Thus, teacher absence can sharply increase the effective STR experienced by students relative to the STR calculated using state-level figures on enrollment and number of teachers.

16 The cross-sectional relationship is estimated by regressing village-level teacher absence on the log of district-level per-capita consumption (from the National Sample Survey) in the 2003 survey. Estimates without state fixed effects are larger (and equal −1.17) whereas estimates with state fixed effects are smaller but still significant (and equal to −0.63). Our default estimate is based on using state-fixed effects since cross-state variation in per-capita income is much more likely to be correlated with unmeasured governance quality. Tables are available on request.

17 This is almost surely a lower-bound estimate because in many cases it is easy for a teacher who may not have been teaching to pick up a book and look like he or she is actively teaching when it is known that someone is visiting the school (see Muralidharan and Sundararaman (2010) for evidence documenting this).

18 Note that teachers sign the log-books when they come in and there is typically no roll call where a head teacher records them as absent if they are not in school at a given time. Thus, the log-books record ‘presence’ rather than ‘absence’. This may explain the higher recorded presence on the previous day than on the day of the visit, since teachers arriving late will sign themselves as present though they may not have arrived during the time the enumerators reached school. It is also not uncommon for teachers to retrospectively sign log-books recording themselves as ‘present’ on days that they were absent.

19 Another way of interpreting the specifications is that the one with no fixed effects is using all the variation in the nationwide changes over time in left and right-hand side variables, and the ones with state and district fixed effects are estimated using within-state and within-district variation in the changes respectively.

20 However, note that the introduction of teacher recognition schemes appears to be correlated with lower teacher absence; with a significant negative correlation in four of six specifications (Columns 1–4) and unchanged point estimates (though not significant) in the other two (Columns 5–6).

21 Doing so does not deviate from the norm in the context of rural Indian government-run primary schools because our data show that close to 80% of schools practice multi-grade teaching (where one teacher simultaneously teaches students across multiple grades at the same time in the same classroom) in any case.

22 In further analysis, we find support for both these channels. Decomposing changes in STR into changes in enrollment and changes in number of teachers, we find that the former are positively correlated with changes in teacher absence and the latter are negatively correlated (results available on request). However, we focus our discussion on the STR because the policy goals for teacher hiring are stated in terms of STR, and because changes in the number of teachers are highly correlated with changes in enrollment.

23 We also consider two alternative constructions: “Probability of being inspected in last 2 months” and “Probability of being inspected in last 1 month.” Results are similar and available upon request.

24 However, since we cannot completely rule out this possibility, our policy recommendations use a decision-theoretic approach to expanding school monitoring that accounts for this uncertainty (see Section 6).

25 In addition to the possibility of formal disciplinary action against absent teachers, an additional channel for the deterrence effect of increased inspections on teacher absence may stem from the possibility that inspectors can extract side payments from absent teachers in return for not making a formal adverse entry on their service record (World Bank, 2003). Social norms would make it difficult to ‘extort’ such payments from teachers who are actually present, but it would be much easier to demand a payment from an absent teacher in return for not initiating formal action. Thus, even if the costs of initiating formal disciplinary action are high (and the incidence of such action is low), there may be other informal channels through which more frequent inspections serve as a disincentive for teacher absence. We also test to see if increased inspections are only correlated with reduced absence rates for contract teachers (who have less job security), and find that this is not the case. Increased inspections are significantly correlated with reductions in absence for both regular and contract teachers, and there is no significant difference between the two.

26 This module was designed to complement the school surveys by allowing us to create quantitative measures of district-level education governance. Unfortunately, the non-completion rate for these interviews was very high (over 40%) due to non-availability, and non-response of district-level administrators. Since this non-response is clearly not random, we do not use the quantitative measures in regressions. Nevertheless, important qualitative insights can be obtained from these interview transcripts. These results are summarized in a companion policy report (Centre for Policy Research, 2012).

27 Regressions in Table 6 include controls for changes in all characteristics reported in Table 4, but those coefficients are not shown since that is not the focus of our analysis. Results are similar without the controls.

28 Duflo et al. (2012) show experimentally that lower teacher absence raises test scores, while Muralidharan (2012) shows this in value-added estimates with five years of annual panel data on test scores in the state of Andhra Pradesh matched with the absence rate of the teacher of each student that year. Das et al. (2007) show that high teacher absences in Zambia lead to significantly lower student test score gains. See (Muralidharan, 2013) for a review of this evidence with a focus on India.

29 Note that teacher absence per se does not entail an economic cost because it is simply a transfer of resources from the tax payer to absent teachers (just like leakage is a transfer from taxpayers to corrupt officials). Thus, the economic cost of teacher absence is the long-term cost of poor service delivery (such as lower long-term human capital and earnings). In practice, it is difficult to quantify these costs. Further, since voters and tax-payers place intrinsic value on not wasting their money, there is considerable policy interest in reducing leakage. This is why the corruption literature has typically focused on estimating ‘leakage’ both to quantify corruption, and to use as a metric to study the impacts of interventions to reduce it.

30 Detailed state-level figures on the number of teachers and their average salaries are presented in Table A7. We augment the salary figures by 10% to reflect government contributions to pensions. This is a conservative estimate since most of the older cohorts of teachers are covered by a more expensive defined benefits pension plan. No adjustment is made for medical benefits. We use the total number of primary school teachers by state because the DISE data provides only the total number of teachers by state, and not the urban-rural breakdown. Moreover, Kremer et al. (2005) report very similar teacher absence rates across urban and rural schools (24.8% versus 26.3%) and so we use the 2010 state-level teacher absence rates for our calculations, with the caveat that these are for rural areas.

31 We use DISE data on the number of schools in each state to calculate the number of supervisors who will be required to increase the probability of inspections in a 3-month interval by 10 percentage points. The cost estimates are conservative and assume that the salary costs are double that of a teacher and that the travel costs are equal to 80% of a full months' salary (which is higher than the typical travel and daily allowance provided to education department employees to travel to/from a village to district headquarters).

32 Note that the estimated cost of achieving a given ESTR reduction through hiring more teachers is higher than the cost of achieving a proportional STR reduction, because our estimates suggest that reducing STR will increase the absence rates of the existing teachers (we use the most conservative estimate from Table 4 for this calculation). In other words, this figure accounts for the fact that we estimate that reducing STR is correlated with increased teacher absence rates, suggesting that increased spending on hiring teachers is correlated with an *increase* in inefficiency as seen from the discussion in Section 4.2.1.

33 Note that the economic benefit to reducing teacher absence may also include a reduction in student absence. However, if we assume that any reduction in student absence in response to a lower ESTR will be the same regardless of the specific policy by which the reduction in ESTR is brought about, then our assessment of the relative cost effectiveness of different policies to reduce ESTR will not be affected.

34 The largest education experiments to date that we know of have been conducted over five districts in one state of India (Muralidharan and Sundararaman, 2010, Muralidharan and Sundararaman, 2011, Muralidharan and Sundararaman, 2013). While these experiments feature random assignment in representative samples of schools (in a state with over 80 million people), they still come from just one state, compared to the estimates in this paper that use panel data from 190 districts across 19 states.

35 Muralidharan et al. (2016) is an example of just such an experimental evaluation, in the context of an ambitious initiative by the Government of Andhra Pradesh (AP) to improve governance in public welfare programs through biometric payments infrastructure. Working with the government of AP, they randomize the rollout of the new biometric payments infrastructure over a potential universe of 20 million beneficiaries, and estimate that the program reduced ‘leakage’ in the rural employment guarantee scheme by an amount that was nine times the cost of the program. Interestingly, this effect is of a similar magnitude to the returns that we estimate to investing in better monitoring of teachers in this paper.
